# The Effect of Biodentine Maturation Time on Resin Bond Strength When Aged in Artificial Saliva

**DOI:** 10.1155/2020/8831813

**Published:** 2020-10-22

**Authors:** Ruba M. Mustafa, Suhad J. Al-Nasrawi, Abtesam I. Aljdaimi

**Affiliations:** ^1^Department of Conservative Dentistry, Faculty of Dentistry, Jordan University of Science and Technology, Irbid, Jordan; ^2^Department of Conservative Dentistry, Faculty of Dentistry, University of Kufa, Najaf, Iraq; ^3^College of Dentistry and Oral Surgery, Alasmarya University, Zliten, Libya

## Abstract

Biodentine is a calcium silicate cement (CSC) that has been broadly applied in vital pulp therapy. The quality of the Biodentine-composite bond has a significant effect on the longevity of the definitive restoration. The aim of this study is to investigate the shear bond strength (SBS) between Biodentine and composite restoration at different maturation times of Biodentine aged in artificial saliva. Fifteen Biodentine discs were allocated into three groups (*n* = 5) based on the timeframe of performance of composite restoration: immediate (after 12  min), after 14 days, and after 28 days of Biodentine maturation. Total etch and rinse adhesive system and bulk-fill regular resin composite were used. The shear bond strength and the failure pattern were assessed. One-way ANOVA with the Bonferroni post hoc test was applied for statistical analysis at *p* < 0.05. The highest (32.47 ± 8.18 MPa) and the lowest (4.08 ± 0.81 MPa) SBS values were recorded for 14 days and 12 min groups, respectively. Significant statistical differences were reported among the groups, and a high statistically significant difference was found between the immediate group and the other groups. Adhesive failure patterns were evident in all groups. More clinically acceptable bond strength between the Biodentine and overlaid composite restoration is at 14 days after Biodentine maturation. Delaying the coverage of Biodentine later than 14 days may significantly reduce the SBS. Using the artificial saliva as an aging medium may affect the SBS between Biodentine and composite material.

## 1. Introduction

Biodentine is a novel repair material with promising characteristic features such as biocompatibility, bioactivity, biomineralization capacity [1, 2], antibacterial activity [3], and great sealing ability [4]. In comparison to its precursor (MTA: mineral trioxide aggregate), it has a shorter setting time, higher viscosity, easier application [5, 6], and lesser discoloration [7], with improved physical characteristics [1, 8]. It has been introduced as an efficient bioactive dentine substitute [2] due to its similar mechanical properties to those of dentine [9]. It has been broadly applied in dental treatment as retrograde filling, treatment of perforation, vital pulp therapy as direct and indirect pulp capping to maintain its vitality, protecting the dentine-pulp complex [10], and regenerative endodontic approach of treatment for immature necrotic teeth [11]. However, because of the Biodentine brittleness, low wear resistance, and unsuitable aesthetic, a laminate restoration is required by an overlaid layer of the dental composite [12].

The quality of the Biodentine-composite bond has a significant impact on the longevity of the final restoration [13]. The experimental data regarding the time after which the Biodentine should be overlaid with the composite are rather controversial, and more research is required to define the proper time. Some authors stated that the final restoration could be executed immediately following the Biodentine placement [14]. Others argue that the final filling is best to be performed after at least 72 h [15] or weeks [13, 16]. To our knowledge, however, it was noted that aging medium mentioned in the literature was either distilled water [13, 16] or 100% relative humidity [5, 14, 17, 18], which is dissimilar to the clinical condition where the material is exposed to saliva. This is the first study that tried to simulate the clinical *in vivo* condition by aging the Biodentine using artificial saliva, especially, the type of aging medium that has an effect on cement surface behaviour. Moreover, it is assumed that the integrity and stability of the Biodentine surface are essential to achieve good adhesion with overlaying restoration.

The null hypothesis states that there would be no statistically significant effect of Biodentine aging in artificial saliva on shear bond strength (SBS) between Biodentine and overlaid composite.

## 2. Materials and Methods

### 2.1. Sample Size Calculation

Sample size was calculated using a sample size calculator [19]. The study power was set at 80% with a margin of error of 5%. The expected mean difference (∆) was 12.67, and the standard deviation of difference (*σ*) was 8.488 according to previous study findings with a similar design [18]. This resulted in a minimum sample size of 4 samples for each group and 5 samples in case of 10% drop out.

### 2.2. Sample Preparation

Fifteen standardized cylindrical Teflon (polytetrafluoroethylene) moulds were designed with a central hole of 2 mm in height and 5 mm in diameter. Each cylinder was placed on a glass slap. Biodentine material (Septodont, Saint-Maur-des-Fossés Cedex, France) was prepared by adopting the manufacturers' instructions. Then, it was incrementally placed inside the holes and condensed with the aids of an amalgam carrier and condenser. Each filled mould was covered with another glass slab to ensure that the cement would set facing a smooth and flat plane to establish standardization of the disc surface.

After setting (12 min), the Biodentine discs were assigned randomly into three groups (*n* = 5) depending on the time of composite application after Biodentine maturation. The groups were as follows: immediate application (12 min), after 14 days, and after 28 days of Biodentine maturation.

Each specimen was individually immersed in artificial saliva at 37°C. The artificial saliva was prepared according to Alshali et al.'s study [20]. After the assigned aging time, about a 3 mm circular area in the central part of the top surface of each the Biodentine disc was treated by a two-step etch-and-rinse system of adhesion (AdperTM Single Bond, 3M Oral Care, St. Paul, MN, USA). This treatment includes acid etching by 35% orthophosphoric acid gel (Scotchbond Etching Gel, 3M Oral Care, St. Paul, MN, USA) for 15 s and rinsing with distilled water for 10 s; after that, each sample was dried with a gentle airstream for 10 s. The adhesive bonding resin was applied and light cured using the Optilux light-curing unit (Optilux 501, USA) with an intensity of 620 mW/cm^2^ for 10 s after exposure to a gentle airstream.

Slices of a plastic tube with 2 mm in height and an internal diameter of 3 mm were placed on the bonded areas. Bulk-fill regular resin composite (Tetric EvoCeram®, Ivoclar Vivadent AG, Schaan, Liechtenstein) was condensed into the tube and polymerised for 20 s with approximately 1 mm distance between the tip of the light-curing unit and the specimen surface. After the composite application, the samples were stored in artificial saliva for 30 days at 37°C [13]. The compositions of investigated materials are summarized in Table 1.

### 2.3. Shear Bond Strength Test

After 30 days' storage of each group in artificial saliva at 37°C, the specimen was fixed on the metallic holder in the SBS testing instrument (Zwick/Roell Z020 Testing Instrument, Zwick GmbH & Co. KG, Ulm, Germany). The stainless steel chisel was oriented perpendicularly to the adhesive interface between Biodentine and composite material. The chisel was utilized to inflict a compression load at 0.5 mm/min crosshead speed till debonding occurred [14].

The maximum load of fracture was reported in Newton (N), and then SBS values were determined in megapascal (MPa) using the following equation:

(1) $$\begin{array}{c} \text{strength of shear bond}=\frac{\text{fracture load}\left(\text{N}\right)}{\text{adhesion surface area}\left(A\right)}. \end{array}$$

The SBS was converted from force to pressure (MPa) using the following equation:

(2) $$\begin{array}{c} \text{pressure}\left(\text{MPa}\right)=\frac{\text{fracture force}\left(\text{N}\right)}{\text{area}\left(A\right)=7.065\;{\text{mm}}^{2}}. \end{array}$$

### 2.4. Internal Examination

The debonded surfaces were examined by using a stereomicroscope (MEIJI Techno Co. Ltd., Tokyo, Japan) at 40x magnification to categorize the failure pattern into one of the following types: (a) adhesive type of failure at Biodentine-composite interface; (b) cohesive type of failure within Biodentine or composite material; (c) mixed type of failure of both types. Single-blind observer assessed the failure mode of all the interface surfaces for all samples.

## 3. Statistical Analysis

The data were processed by SPSS software version 20 (IBM Corporation, New York, USA). Firstly, data were tested for normality by Shapiro–Wilk's test. Then, means of SBS of different cement aging groups were tested for the presence of statistically significant difference utilizing one-way analysis of variance (ANOVA), and then the Bonferroni post hoc statistical test for multiple comparisons (*p* ≤ 0.05) was performed.

## 4. Results

The descriptive statistics for the SBS (MPa) for the tested groups are displayed in Table 2. The 14 days' group showed the highest bond strength (32.47 ± 8.18 MPa), while the immediate (12 min) group showed the lowest bond strength (4.08 ± 0.81 MPa). The one-way ANOVA test marked a significant difference between the tested groups, which was specified by the Bonferroni post hoc test. Accordingly, a significant difference was demonstrated between 14 days and 28 days, with a high significant difference between the immediate (12 min) group and other groups (Table 2).

**Figure 1:**
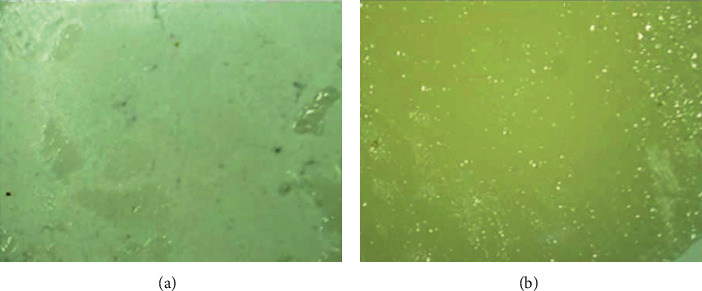
Stereomicroscopic images under 40x magnifications for bond failure. (a) Example of the adhesive failure where the debonded surface of the biodentine substrate (central area) is regular and free of any remnants of composite restoration (adherent) (it was a comparable finding for all examined surfaces of all tested groups). On the outside of the deboned surface, remnants of adhesive agents can be seen. (b) The composite debonded surface (adherent) is regular and free of any remnants of Biodentine (substrate).

Regarding the failure mode of the debonded surfaces (interfaces) between composite and Biodentine, the adhesive failures were exhibited in all samples of all groups regardless of the timing of composite material application over the Biodentine. The Biodentine (substrate) debonded surface was regular, smooth, and free of any remnants of the composite restorative material. At the same time, the composite (adherent) debonded surface was regular, smooth, and free of any remnants of Biodentine (Figure 1).

## 5. Discussion

Biodentine is a bioactive dentine substitute material [2] that has been recommended for coronal, radicular restorations and in vital pulp therapy [8]. Biomimetic materials have been introduced in order to obtain a remineralizing effect on both dentine and enamel, showing promising results in terms of bond strength and tissue microhardness [21]. Strength of the bond between a final filling material and the underlined Biodentine is one of the most critical influences affecting the quality and success of the final restoration. At present, there is limited evidence in the published work on the effect of Biodentine aging in artificial saliva on SBS between the cement and the overlaid adhesive restoration. Regarding the aging medium, in order to simulate the clinical intraoral condition, artificial saliva rather than distilled water or 100% humidity was used, and this is the first time where the artificial saliva was applied as a storage medium for Biodentine laboratory aging. In fact, the type of aging medium has an impact on the surface behaviour and physical properties of the cement. In relation to that, previous studies reported that Biodentine disintegration was less in body fluid or phosphate-buffered saline (PBS) compared to distilled water [22]. In addition, it was proved that using the body synthetic fluid as a storage medium in laboratory studies might result in reduction of microleakage [23] and surface porosities [24] as well as surface microhardness enhancement.

The stated null hypothesis was rejected since there is a significant effect of the Biodentine aging in artificial saliva on the bond strength to composite restoration. In the present research, the strength of the tested shear bond recorded the lowest value after an immediate application of composite and then increased to reach the highest value after 14 days; after that, it showed a reduction as it reached 28 days.

Regarding the Biodentine setting maturation, it undergoes an initial setting reaction which takes about 12 min after mixing of the components, forming a hydrated calcium silicate gel, which has retarded the physicomechanical qualities [25]. A superficial setting is reached at this stage. Therefore, lower SBS values were acquired in the early placement of overlaid composite in comparison to the delayed Biodentine aging group as the composite curing contraction might stress the fragile cement in this early sensitive stage causing premature failure.

With time, Biodentine maturation continues where crystallization of the calcium silicate hydrate gel can last for 14 °days up to one month. The bulk setting is accomplished at this time, enhancing physicomechanical qualities [25]. This might justify the development of bond strengths over time from 12 min to 14 days.

The present results agree with that of Sultana et al.'s study findings [16] as the placement of the composite restoration immediately over the Biodentine displayed the lowest values, but considering the delayed placement (28 days), the current study results disagree with them as they recorded a continuous increase in the strength of the shear bond between the two materials. Although they stored the samples in distilled water which is expected to increase the disintegration of the Biodentine cement, the SBS increased with time because prestorage bonding of their samples was providing a barrier against Biodentine hydrolysis. Our finding regarding the continuous decrease of SBS as it reached 28 days can be explained by the recorded marked increase in solubility of the Biodentine over time [26]. Some materials might be stable after setting but disintegrated with time; the longer the time period, the more the degradation [27]. It has been hypothesized that, for a bioactive material liberating calcium ions to induce a biologic influence, it has to solubilize and disassociate from fully hardened material, hence resulting in disintegration [28].

Current results agree with Hashem et al.'s study findings [13]. They demonstrated that the immediate covering of the cement resulted in weak adhesion. After that, the SBS increased to reach the highest values after 24 h and then decreased to reach comparable values over 6 m. Their result might reflect the early disintegration of Biodentine caused by the water storage after 24 h which reached an equilibrium over 6 m. The present findings of having the highest value of SBS at 14 days might be explained by the late Biodentine disintegration using artificial saliva as a storage medium. After reaching 28 days, the SBS then reduced as the surface degradation of the exposed material was still high as a result of long-term aqueous storage.

It was stated that the highest microhardness value for the Biodentine material was achieved after two weeks' storage [25], and as the SBS was positively proportional with the microhardness [29, 30], the improved bond strength is expected. Kaup et al. [31] recorded a significant rise in the SBS comparing 2–7 days. Furthermore, Bachoo et al. [25] stated that the initial phase of setting reaction of the Biodentine cement takes around 12 min, but the final maturation takes about 14–30 days. In view of that, the setting reaction of the material may influence the strength of the bond between the Biodentine cement and the overlaid composite. In addition, the material rigidity may affect the bond strength interpretation [31]. In relation to that, as the setting reaction for Biodentine and calcium silicate cement (CSC) can continue up to one month [25, 32], the samples were stored in artificial saliva for 30 days after a composite application to ensure the complete maturation of Biodentine before the SBS testing for the interface bond. This will reduce the bias which can result from cohesive fractures within the incompletely matured Biodentine during the SBS testing [31].

Regarding postcomposite application storage, only one study [13] did 30 days' storage in water for the samples after the composite application. Hashem et al. found that SBS was not significantly improved between 5min and 14 days of composite application [13]. On the other hand, the current study findings showed significant improvement in SBS between 12 min and 14 days. This might be related to using the artificial saliva as an aging medium, which is more clinically relevant, rather than water. The previous studies found the positive effect of artificial saliva as a storage medium on the physical properties of the Biodentine and CSC materials compared to water storage such as solubility, porosity, microhardness, and microleakage [22–24, 33], which can be associated with SBS.

Numerous techniques have been used to examine the bond strength between different restorative materials [34]. Among them, the SBS test which is easily applied and has the advantage of the ability to assess brittle materials [13, 34, 35]. This testing method could reduce the pretest stressing factor caused by sample sectioning, which is required in the other types of the SBS test [35]. In view of the mentioned advantages, the SBS test was applied in this study. Samples of the current study were etched by following the Cengiz and Ulusoy [36] recommendation. They stated that Biodentine etching and rinsing might improve adhesion of composite to Biodentine. In addition, an adhesive system was applied using the total-etch technique as recommended by Hashem et al. [13].

Regarding the possible effect of acid etching, it is expected that the exposure of Biodentine cement to an acid with low pH could disturb the chemical setting of the material by affecting the hydration of tricalcium silicates impairing the setting cement's microstructure [37–39]. On the other hand, the acidity of the etching might be buffered by the Biodentine alkalinity, reducing its effect. Cammilleri [40] examined the effect of acid etching on Biodentine. Although structural and chemical alterations were recorded in the etched cement in comparison to nonetched cement, Biodentine hardness did not get affected.

Concerning the mode of failure, the adhesive failure was noticed in all samples, which agrees with Tulunbaci et al. [41] and disagrees with other studies where some cohesive failures were recorded [13, 14]. The recorded cohesive failures in some samples in previous studies might result from the uneven stress distribution intensifying in the substrate resulting in a premature failure in advance to the interface itself [42]. This is an inherent complication accompanying with SBS testing where raised tensile stress initiates below the site of load application while compressive stress generates on the opposing side [35, 43]. Furthermore, the applied bonding layer might extend beyond the tested area changing the way of stress is distributed during testing which can affect test outcomes since the fractured zone is bigger than the interfacial zone assigned to calculate the SBS [35, 44, 45].

It was reported that bond strength at a range from 17 to 20 MPa might be essential to sufficiently encounter contraction forces and create a restoration with gap-free margins [46, 47]. In view of the current results, it is recommended to postpone the placement of the composite restoration to 14 days after the Biodentine initial setting.

Comparing the current study results about SBS with other studies, data are quite difficult because of the wide heterogeneity of tested restorative materials, application times, storage periods, storage medias, and adhesive systems. Nonetheless, there are scarce studies about storage media. As a result, further investigations are recommended for a better understanding of artificial saliva as a aging medium on bond strength. The effect of aging media such as artificial saliva with fluoride on SBS between overlaid composite and Biodentine is a suggested topic for future research.

The main limitations of this laboratory study are the fact that the in vitro studies generated SBS findings should be interpreted with caution to the clinical situation. Therefore, future clinical trials and in vivo studies are highly important for better understanding of the bonding mechanism to Biodentine in the oral environment. It is worth mentioning that the present study showed higher bond strength values after the storage in artificial saliva. However, also the thermal cycles in the oral cavity could have an effect on adhesion values [48]. Therefore, further studies are needed in order to test this variable.

## 6. Conclusion

Within the limitation of this in vitro study, it can be concluded that there is a significant effect of Biodentine aging in artificial saliva on SBS bond to composite resin. The shear bonding strength of bonding composite to Biodentine was significantly reduced at the Biodentine primary setting phase or after prolonged time of exposure to the oral environment. The best time for placement of an overlying composite restoration is at 14 days. However, delayed placement of composite up to 28 days significantly affects the SBS.

## Figures and Tables

**Table 1:** Chemical composition and manufacturer information for tested materials.

Materials name	Materials type	Composition	Manufacturer
Biodentine™	Calcium silicate-based material	Liquid: water-reducing agent, water, and calcium chloride Powder: tricalcium silicate, dicalcium silicate, calcium carbonate, zirconium oxide, iron oxide, and oxide ﬁller	Septodont, St Maure des Fossés, France
Adper Single Bond 2 adhesive	Total etch-rinse/two-step adhesive system	BisGMA, a methacrylate functional copolymer dimethacrylates, HEMA, water, ethanol, colloidal filler, and photoinitiator	3M Oral Care, St. Paul, MN, USA
Scotchbond Etching Gel	Acid etching	35% orthophosphoric acid gel	3M Oral Care, St. Paul, MN, USA
Tetric EvoCeram® Bulk-Fill	Light cure nanohybrid composite resin restoration	61% (vol.) as filler (barium aluminium silicate glass) and 17% “isofillers” which compose dimethacrylates, glass filler, and ytterbium fluoride. TEGDMA, UDMA, and BISGMA	Ivoclar Vivadent AG, Schaan, Liechtenstein

HEMA: 2-hydroxyethyl methacrylate; TEGDMA: triethylene glycol dimethacrylate; UDMA: urethane dimethacrylate; BisGMA: bisphenol A glycidyl dimethacrylate.

**Table 2:** The mean shear bond strength in MPa, standard deviation (SD), medium, maximum, and minimum for all tested groups.

Timing of composite restoration application over Biodentine group (*n* = 5)	Mean (MPa)	SD	Medium (MPa)	Maximum (MPa)	Minimum (MPa)
Immediate application (12 min)	4.08^a^	0.81	4.195329	4.939844	2.755839
After 14 days	32.47^b^	8.18	33.48195	41.82873	19.28096
After 28 days	23.35^c^	3.53	23.76079	26.862	18.58316

Mean bond strength values sharing the different superscript letters were statistically significantly different (*p* < 0.05).

## Data Availability

The data used to support the findings of this study were supplied by the corresponding author. Requests for access to these data should be made to Ruba Mustafa (rmmustafa@just.edu.jo).
